# Study of the Cold Curing Characteristics of Isocyanate-Modified Asphalt

**DOI:** 10.3390/ma17051048

**Published:** 2024-02-24

**Authors:** Changhong Zhou, Mingli Peng, Xue Yang, Yating Qi, Bin Xu

**Affiliations:** 1 School of Architecture and Transportation Engineering, Guilin University of Electronic Technology, Guilin 541004, China; czhou@guet.edu.cn (C.Z.); 15179619121@163.com (M.P.); 2 Key Laboratory of New Infrastructure Construction in the Transport Sector, Education Department of Guangxi Zhuang Autonomous Region, Guilin 541004, China; 3 School of Civil Engineering, Hebei University of Architecture, Zhangjiakou 075000, China; yangxue19930817@163.com; 4 School of Transportation and Logistics, Dalian University of Technology, Dalian 116024, China; qiyating2009@163.com; 5 Research Institute of Highway Ministry of Transport, Beijing 100088, China

**Keywords:** asphalt mixture, isocyanates, cold curing, molecular dynamics simulation

## Abstract

Isocyanate esters are widely recognized for their superior curing capabilities. Leveraging this attribute, the current research formulated a modified cold-mixed asphalt blend using 4,4′-methylene diphenyl diisocyanate (MDI). Tests and analyses of the MDI-modified asphalt with varying inclusion percentages of MDI revealed that a mixture containing 15% rock asphalt and 15% MDI-modified asphalt exhibited a more balanced, comprehensive performance. We also conducted an examination of the role and properties of MDI in asphalt modification using molecular dynamics simulations. The cold-curing properties of MDI-modified asphalt as compared to petroleum asphalt were evaluated based on its density, free volume analysis, cohesive energy density, and glass transition temperature. Implementing the LB-13 gradation—a cold-mixed asphalt gradation with a nominal particle size of 13.2 mm recommended by Chinese specifications—we prepared MDI-modified cold-mixed asphalt and carried out analyses of its mechanical characteristics, high-temperature performance, and water damage resistance. The results demonstrated that MDI-modified asphalt showcases excellent ductility, flexibility, and aging resistance, surpassing the performance of petroleum asphalt. The stability, high-temperature rutting, and water damage resistance of the MDI-modified cold-mixed asphalt exceeded the requirements for hot-mixed asphalt. This research provides theoretical and experimental support for isocyanate ester applications in asphalt engineering, presenting significant value for practical engineering applications.

## 1. Introduction

Asphalt concrete [1,2,3], a major road construction material, frequently encounters issues like degradation, cracking, and fatigue under harsh climatic conditions and traffic pressure [4,5]. To enhance road durability and safety, researchers have been exploring new methods and materials to augment the performance of asphalt concrete.

One such method is asphalt modification, and within this field, methylene diphenyl diisocyanate (MDI) is gaining attention for its potential as a modifier. The application of MDI for asphalt chemical modification offers dual benefits. Firstly, the application of MDI enhances the engineering performance of asphalt materials, thereby reducing the damage caused by environmental factors and loads to the pavement. Additionally, as a cold-mix modifier, MDI can form high-performance cold-mix asphalt mixtures, thereby curbing carbon emissions generated during the construction process. As MDI exhibits a diverse reactivity allowing it to react with the hydroxyl groups within asphalt molecules [6,7], the cross-linking structure formed due to the reaction between MDI and hydroxyl groups enhances the water resistance of asphalt, reducing the impact of moisture on the erosion of pavement structures. Therefore, it considerably enhances asphalt properties. Feng et al. constructed and confined MDI-modified asphalt models through a molecular dynamics simulation to investigate the effect of the macromolecules of the nucleophilic addition reaction on the modification [8]. Li et al. combined physical–chemical characterization and quantum chemical calculations based on Density Functional Theory (DFT) and facilitated a multiscale interpretation of the interactions and cross-linking behavior of MDI-modified asphalt molecules [9]. Despite these studies, there is still a necessity for further research to elucidate the molecular-level modification mechanism.

With the development of micro-scale numerical simulation technology, molecular simulation can visualize the morphological changes and chemical reactions of materials at the molecular level [10]. Molecular dynamics (MD) simulations are able to improve the understanding of these phenomena by combining theoretical approaches and computer technology, and enhance the investigation of the principles of material property improvement [11,12,13,14]. As a result, more and more researchers are applying MD in the field of polymer–asphalt compatibility. Su [15] and Yu [16] established a model of zinc oxide and carbon nanotubes (CNTs) and used the MD method to investigate their effects on the molecular adsorption and diffusion capacity of SBS-modified asphalt. Meanwhile, researchers have calculated the solubility parameters of asphalt and polymer modifiers based on MD results and predicted the compatibility between polymer modifiers and asphalt [17,18]. In addition, dos Santos [19] conducted dissolution experiments and modeling of asphalt samples in polar and non-polar synthetic mixtures. Ahmadi [20] used molecular dynamics (MD) simulation to simulate the rheological behavior of asphalt at different temperatures. At present, molecular dynamics (MD) simulation is becoming an efficient analytical tool for predicting the performance of modified asphalt. However, there are few existing reports on the compatibility between isocyanates and asphalt using this method.

Cold-mix asphalt mixtures (CMA), regarded as crucial pavement materials for reducing energy consumption and decreasing carbon emissions, have attracted global attention. Chavez-Valencia [21] utilized polyvinyl acetate as a chemical additive in the preparation of cold-mix asphalt mixtures. After modification with polyvinyl acetate, the cold-mix asphalt mixtures exhibited improved compressive strength. Geng L. [22] developed cold-mix asphalt for pothole repair using a simple mixture of cooking waste oil and diesel as a diluent and assessed its performance in the laboratory. Sena Neto [23] prepared cold-mix asphalt mixtures using construction and demolition waste, and conducted relevant analyses. MDI also serves as an effective modifier for cold-mix asphalt mixtures, leading to research and development based on its cold-curing mechanism [24,25].

This study focuses on addressing the performance issues of cold-mixed asphalt mixtures. Based on molecular dynamics simulation and an analysis of the performance of asphalt mixtures, MDI-modified asphalt was prepared and the compatibility between asphalt and MDI was investigated, which lays the foundation for the development of high-performance cold-mixed and cold-paved asphalt mixtures.

## 2. Experiments

### 2.1. Raw Materials

**Figure 1:**
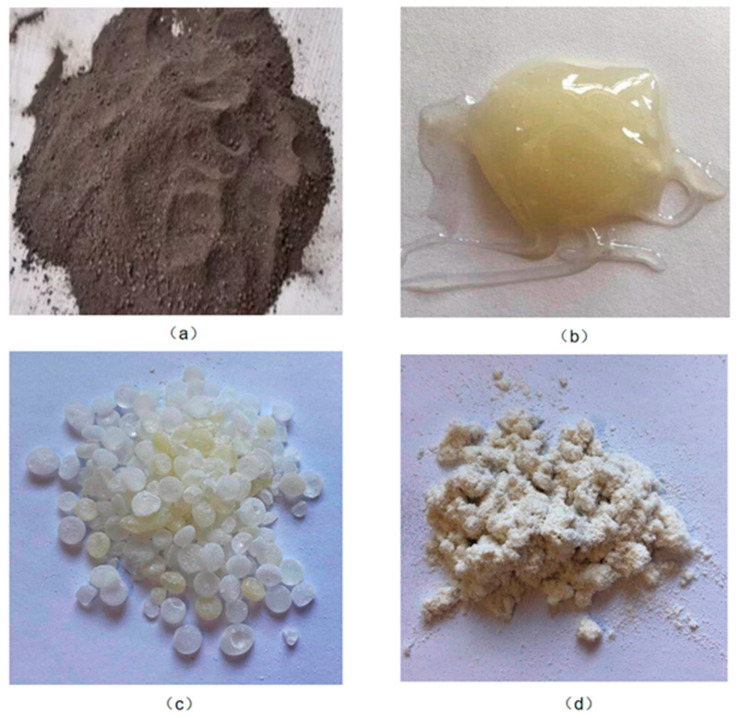
The appearance of some experimental materials. (**a**) Rock asphalt, (**b**) MDI before curing, (**c**) Petroleum resin, and (**d**) Cellulose.

The materials required for the experiments included petroleum asphalt, rock asphalt, MDI (4,4′-methylene diphenyl diisocyanate), petroleum resin, triethylenediamine, ethylene glycol, cellulose, and kerosene, with the appearance of some of them shown in Figure 1. The technical properties of the petroleum asphalt used are outlined in Table 1, as per the methods provided by the Standard Test Methods of Asphalt and Bituminous Mixtures for Highway Engineering (JTG E20-2011) [26]. Rock asphalt was sourced from Buton Island, Indonesia. The other materials were procured from the market.

The technical properties of rock asphalt are shown in Table 2. Since the cured product of MDI has a relatively low elastic modulus, rock asphalt is used to enhance the overall modulus of the modified asphalt, while petroleum resin is utilized to increase the material’s plasticity and compatibility.

MDI is mainly based on poly-isocyanates (-NCO) or other reaction products as the base material; the cold-mixed asphalt developed in this study was mainly based on the wet-curing principle of MDI, which reacts with water in the following process:

$$H_{2}O+\mathrm{OCN}-R-\mathrm{NCO}+H_{2}O\to H_{2}N-R-{\mathrm{NH}}_{2}+{\mathrm{CO}}_{2}$$

(OCN−R−NCO denotes a prepolymer containing a terminal group) (amine)

$$\mathrm{OCN}-R-\mathrm{NCO}+H_{2}N-R-{\mathrm{NH}}_{2}\;\to {\left(-\mathrm{HN}-R-\mathrm{NH}-\mathrm{CO}-\mathrm{NH}-\mathrm{NHCO}-\right)}_{n}\;\;\;(\mathrm{urea}\;\mathrm{bond})$$

The first stage is the initial curing stage, also known as the initial bonding stage. During this phase, MDI primarily undergo a physical curing process rather than a chemical reaction. Typically, most MDI samples do not exhibit an ideal strength during the initial bonding stage, where processes such as solvent evaporation, preliminary cross-linking of adhesive molecules, and crystallization mainly occur [27,28].

The second stage is the final curing stage, the phase where the ultimate strength is achieved. At this stage, the adhesive undergoes a chemical curing process, determining the formation of the final bonding strength. Following the initial curing stage, the -NCO groups in MDI gradually undergo a complete reaction over time and solvent evaporation completes, transitioning the adhesive into its final cured state. This process is a chemical one that drives the adhesive towards its ultimate cured state.

### 2.2. Preparation Method of MDI-Modified Asphalt

**Figure 2:**
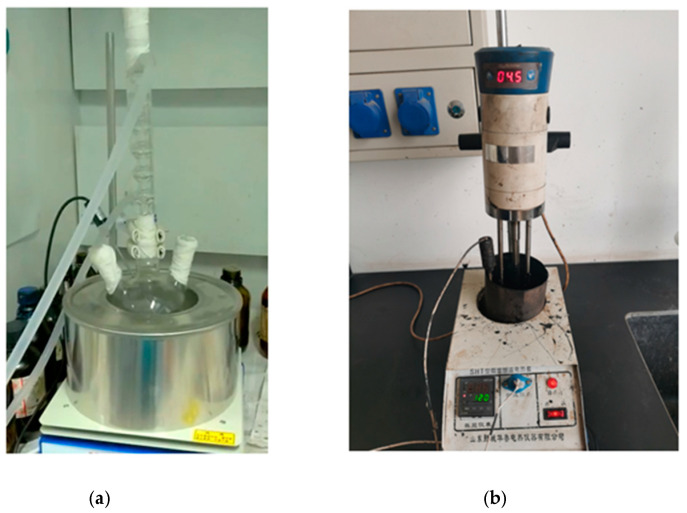
MDI-modified asphalt preparation. (**a**) Constant temperature heating magnetic agitator, (**b**)Adjustable high-speed rotor.

The MDI-modified asphalt can be prepared according to the components specified in Table 3. Firstly, place the asphalt in a flask and heat it within a temperature range of 130 °C to 160 °C in a magnetic agitator with the oil bath (Figure 2a). Heat the rock asphalt mixture for 20 min until it dissolves into the petroleum asphalt. Stir the asphalt solution at 160 °C for 40 min to ensure thorough dissolution and obtain the modified asphalt. Subsequently, add the petroleum resin and vigorously stir the mixture using a rotor for 2 min until fully dissolved. Next, reduce the temperature to a range between 80 °C and 120 °C and introduce kerosene to dilute the asphalt, bringing it to a flowable state for 2 min. Then, further decrease the temperature to a range of 110 °C to 50 °C and sequentially add MDI, coagulant, ethylene glycol, and cellulose. Stir the components using the rotor (Figure 2b) for 2 min to ensure complete dissolution and thorough mixing of each ingredient. Finally, utilize an adjustable high-speed homogenizer for 5 min of shearing to further ensure the thorough mixing of all components.

### 2.3. Technical Properties of MDI-Modified Asphalt

By conducting orthogonal experiments on different contents of MDI, rock asphalt, and petroleum asphalt, we formed three main types of different modified asphalts: (1) MDI-modified asphalt with varying contents of MDI (10%, 15%, 20%) and petroleum asphalt contents; (2) rock asphalt-modified asphalt with varying contents of rock asphalt (10%, 15%, 20%) and petroleum asphalt contents; (3) MDI and rock asphalt-modified asphalt, where we selected one type of MDI-modified asphalt with balanced performance and blended it with different contents of rock asphalt (10%, 15%, 20%) to form MDI-modified cold-mixed asphalt. Their technical properties—penetration value, softening point, and ductility—were tested according to China’s specification JTG E20-2011 [26], and the results are shown in Table 4. Since the MDI modifier is relatively soft and the addition of MDI reduces the viscosity of petroleum asphalt, penetration values for the MDI individually modified asphalt samples could not be effectively measured in the experiment.

From Table 4, the following can be seen:

(1) As the MDI content increases, the softening point of MDI-modified asphalt gradually decreases, while the ductility increases. This indicates that the addition of MDI worsens the high-temperature performance of the asphalt but enhances its low-temperature performance.
(2) The penetration value of modified asphalt is inversely proportional to the amount of rock asphalt added. With an increase in the rock asphalt content, the penetration value of modified asphalt gradually decreases. Additionally, as the rock asphalt content increases, the high-temperature performance of MDI asphalt improves: the penetration value decreases gradually, and the softening point increases. This suggests that the addition of rock asphalt has a positive impact on enhancing the high-temperature performance of the modified asphalt.
(3) Based on the trend of the penetration, softening point, and ductility indicators of MDI-modified asphalt with varying rock asphalt contents, it was found that when the rock asphalt content is 15% and the MDI-modified asphalt content is 15%, the comprehensive performance of the modified asphalt is relatively balanced. Therefore, this study selected a mixture of 15% rock asphalt and 15% MDI-modified asphalt as the research subject.

## 3. Molecular Dynamics Simulation

### 3.1. Molecular Modeling of Asphalt Components and MDI-Modified Asphalt

**Figure 3:**
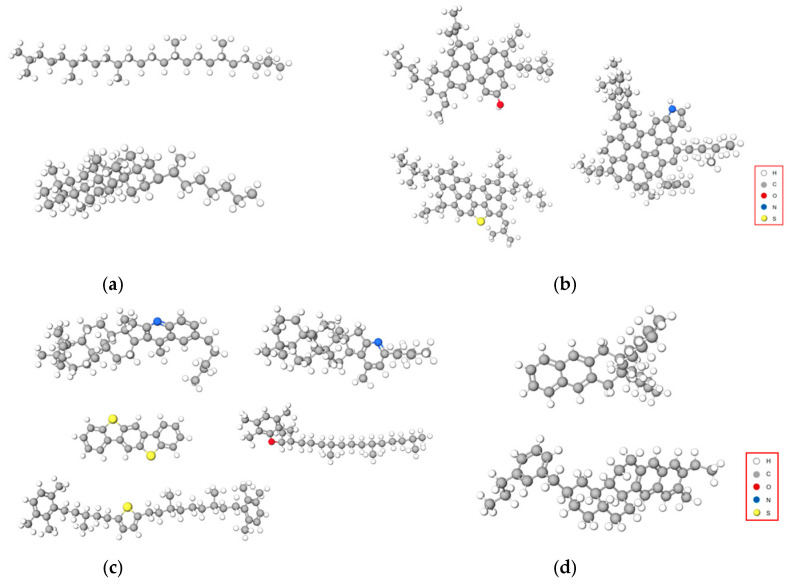
Original asphalt molecular model. (**a**) Saturates, (**b**) asphaltenes, (**c**) resins, and (**d**) aromatics.

**Figure 4:**
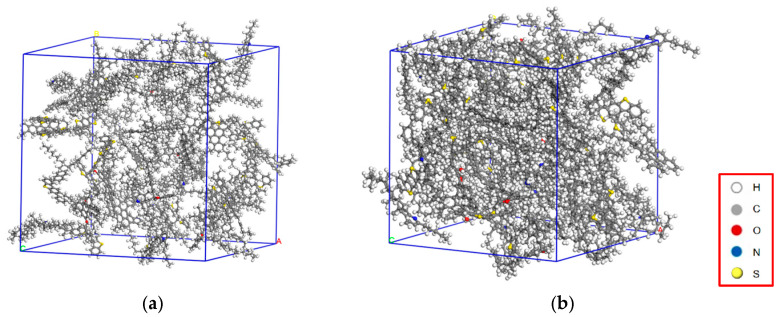
Petroleum asphalt molecular model. (**a**) Initial model of asphalt and (**b**) equilibrium model of asphalt. (Note: A, B, and C in the figure represent the three directional vectors of the unit cell).

Asphalt molecular dynamics simulation is a method that employs computer simulation techniques to study the structure, properties, and behavior of asphalt molecules. This simulation utilizes the principles of molecular dynamics to investigate the interactions, movements, arrangements, and changes among asphalt molecules, exploring their behaviors under different conditions [29,30,31,32]. Due to asphalt being a chemical mixture composed of various hydrocarbons and miscellaneous atoms such as oxygen, sulfur, and nitrogen [33], detailed information regarding its chemical composition is challenging to obtain. Asphalt is classified into four different components based on molecular size, solubility parameters, and selective adsorption–desorption behavior: saturates, asphaltenes, resins, and aromatics. Referring to Li and Greenfield’s proposal [34], this study used a 12-component asphalt model to represent the original asphalt, as shown in Figure 3. The asphalt molecular model assembly was conducted using the amorphous cell module in Materials Studio software (MS, v2017). The initial model and the equilibrium model of asphalt are depicted in Figure 4.

**Figure 5:**
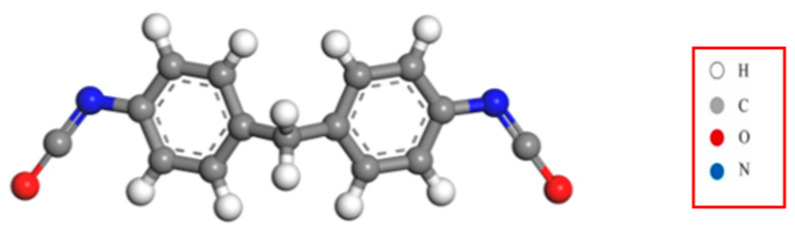
MDI molecular structure model.

**Figure 6:**
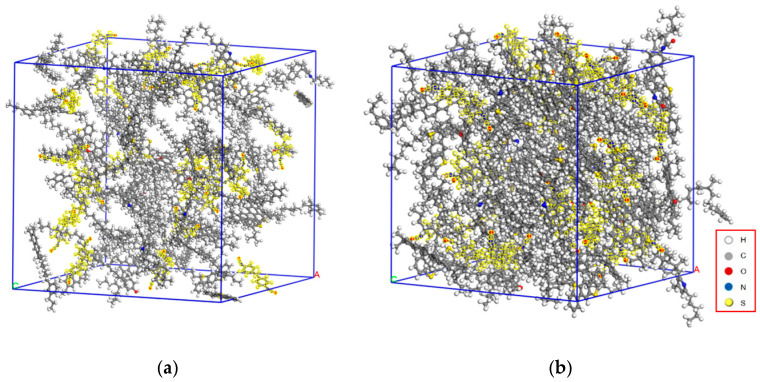
MDI initial and equilibrium models. (**a**) Initial model of MDI and (**b**) equilibrium model of MDI. (Note: A, B, and C in the figure represent the three directional vectors of the unit cell).

MDI (methylene diphenyl diisocyanate) is a polyurethane material synthesized from isocyanates, polyols, and their coordinating agents. Renowned for its exceptional properties, diverse varieties, and extensive applications, MDI stands out among synthetic materials and is one of the fastest-growing materials in use today. In this experiment, a molecular simulation of MDI-modified asphalt was performed employing Materials Studio software to construct models of the MDI molecular structure (Figure 5), the initial MDI model, and the equilibrium state model (Figure 6), as shown below.

### 3.2. Analysis and Discussion

#### 3.2.1. Density Analysis

**Figure 7:**
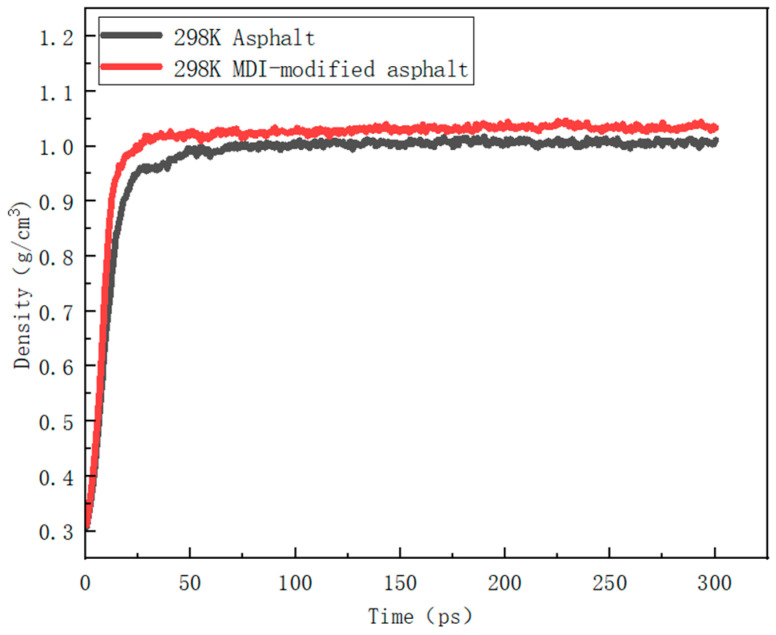
Density variation curve of asphalt and MDI-modified asphalt at 298 K.

The simulated density of the MDI-modified asphalt was first compared with the experimentally obtained density. Experimental density is typically derived by measuring mass and volume. The simulation results aid in assessing the compressibility and deformation behavior of MDI-modified asphalt, offering insights into the material’s compactness. A higher density may indicate a more closely packed molecular structure of asphalt with fewer intermolecular gaps, whereas a lower density might imply a more open structure or increased pore space. Figure 7 depicts the density variation curve between asphalt and MDI-modified asphalt at 298 K.

From the image, it is apparent that the density of MDI-modified asphalt is significantly higher than that of regular asphalt, indicating a more tightly arranged molecular structure and a higher mass of MDI-modified asphalt. The increased density of the MDI-modified asphalt reflects the impact of MDI as a modifier on the asphalt. MDI molecules may undergo chemical reactions or physical adsorption with asphalt molecules, leading to a tighter arrangement between the molecules. This contributes to enhancing the performance of MDI-modified asphalt, improving its compression resistance and durability.

#### 3.2.2. Free Volume Analysis

**Figure 8:**
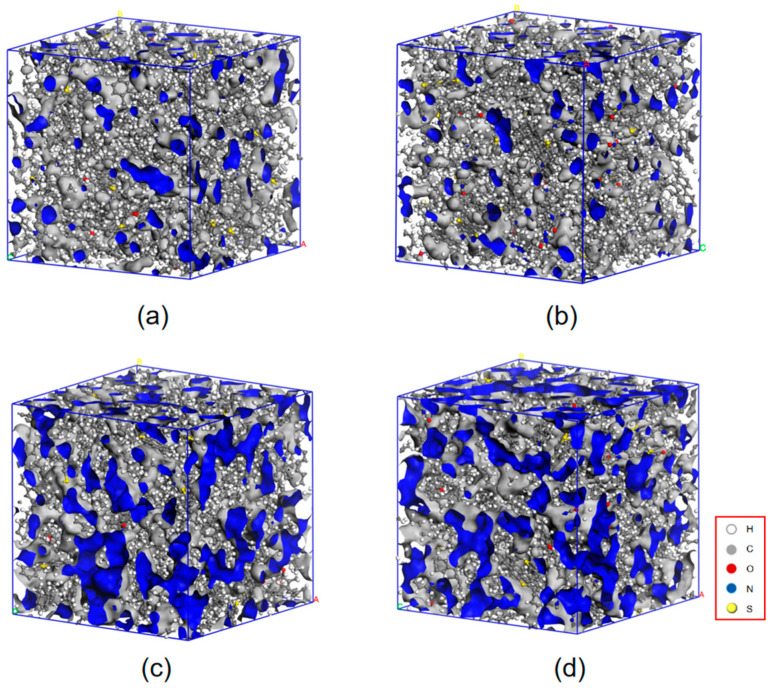
Free volume plots of asphalt and MDI-modified asphalt at 298 K and 400 K. (**a**) Free volume of asphalt at 298 K, (**b**) Free volume of MDI-modified asphalt at 298 K, (**c**) Free volume of asphalt at 400 K, and (**d**) Free volume of MDI-modified asphalt at 400 K. (Note: A, B, and C in the figure represent the three directional vectors of the unit cell. Blue is the free volume not occupied by asphalt).

The free volume fraction represents the proportion of unoccupied space within asphalt molecules. This value describes the internal pores or voids within asphalt molecules, signifying the space not occupied by the actual molecular structure. Changes in the free volume fraction may reflect the flexibility, flowability, or arrangement of asphalt molecules. If the free volume decreases to a certain extent, the asphalt may lose its ability to deform and transition into a glassy state. Molecular models of asphalt at a constant temperature of 298 K, both for regular asphalt (a) and MDI-modified asphalt (b), as well as at 400 K for asphalt (c) and MDI-modified asphalt (d), were simulated using MS software. Free volume plots of asphalt and MDI-modified asphalt at 298 K and 400 K are depicted in Figure 8.

**Figure 9:**
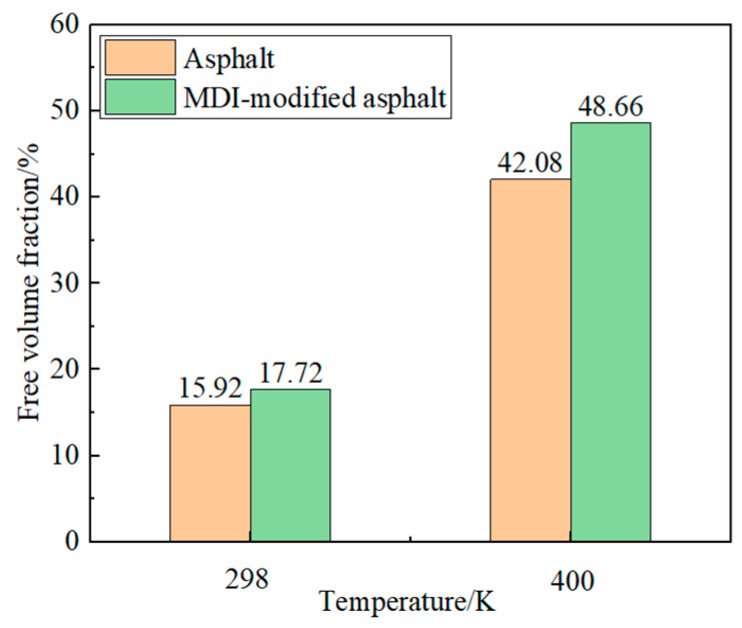
Simulated results of the free volume fraction for asphalt and MDI-modified asphalt.

An analysis of their road performance using the free volume fraction calculation method is shown in Equation (1):

(1) $$\mathrm{FFV}=\frac{V_{f}}{v_{f}+V_{0}}\times 100\%$$

where FFV is the free volume fraction, %; $V_{f}\;$ is the free volume, ${\mathrm{cm}}^{3};$ and $V_{0}$ is the occupied volume, ${\mathrm{cm}}^{3}$. The simulation results of the free volume fraction of matrix asphalt and MDI-modified asphalt are shown in Figure 9.

From the graph, it is evident that the free volume fraction of the MDI-modified asphalt has increased by 1.80% and 6.58% compared to the base asphalt. This indicates that the MDI-modified asphalt has a larger internal free space, facilitating asphalt diffusion, softening, and enhancing its low-temperature performance. At 400 K, both the MDI-modified asphalt and base asphalt experienced a slight increase in the free volume fraction. This outcome is due to molecular thermal movement; asphalt materials tend to soften at higher temperatures, reducing the elastic components and increasing the viscous components, thereby increasing the volume of asphalt molecules that can move freely.

#### 3.2.3. Cohesive Energy Density

Cohesive energy density (CED) is an important thermodynamic property that measures the mutual attraction between molecules and is used to evaluate the intermolecular interactions in isocyanate–asphalt blend systems. Its calculation formulas are shown in Equations (2) and (3):

(2) $$E_{\mathrm{CED}}=\frac{E_{\mathrm{coh}}}{V}$$

(3) $$E_{\mathrm{coh}}=-\langle E_{\mathrm{inter}}\rangle =\langle E_{\mathrm{intra}}\rangle -\langle E_{\mathrm{total}}\rangle$$

where $E_{\mathrm{CED}}$ is the cohesive energy density, $J/{\mathrm{cm}}^{3}$; $E_{\mathrm{coh}}$ is the cohesive force, N; V is the volume of the system, ${\mathrm{cm}}^{3}$; $E_{\mathrm{inter}}$ is the intermolecular energy, J; $E_{\mathrm{intra}}$ is the intramolecular energy, J; and $E_{\mathrm{total}}$ is the energy of the whole system, J.

**Figure 10:**
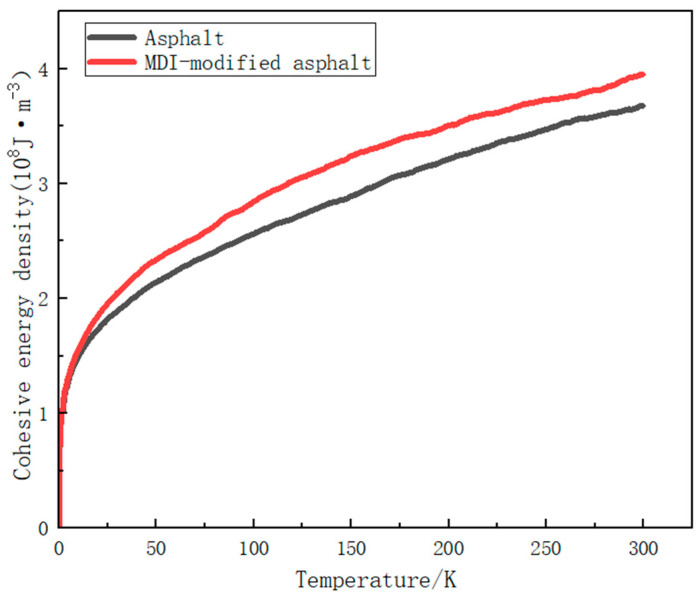
Cohesion energy density variation curves for base asphalt and MDI-modified asphalt.

The simulation results of the cohesive energy density of matrix asphalt and MDI-modified asphalt with the process of a temperature increase are shown in Figure 10.

From the cohesion energy density change curve in Figure 10, it can be observed that the total cohesion energy density of the MDI-modified asphalt is slightly higher than that of the regular asphalt. This indicates that after the solidification process induced by the MDI modification, the internal interactions among asphalt molecules intensify, resulting in a greater bonding strength between the molecules in the MDI-modified asphalt compared to the regular asphalt. This modification enhances the durability and stability of asphalt pavement, augmenting its resistance to deformation and aging. These improved properties contribute to increased pavement stability and longevity, reducing damage and maintenance requirements.

#### 3.2.4. Glass Transition Temperature

**Figure 11:**
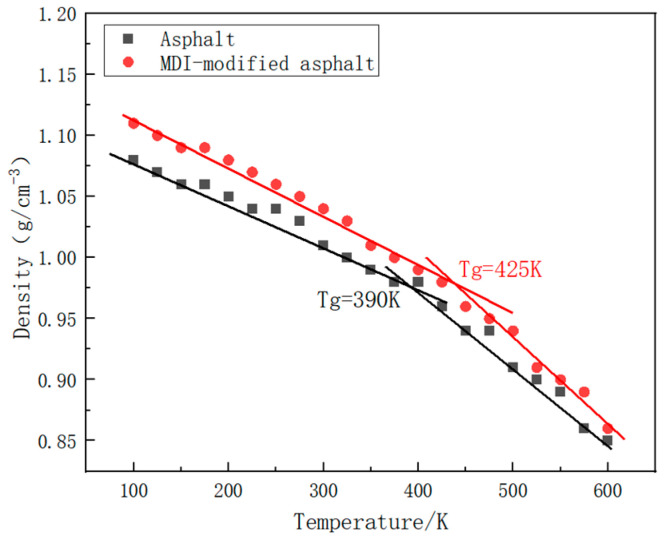
Glass transition temperature diagram of base asphalt and MDI-modified asphalt.

The glass transition temperature ($T_{g}$) is the temperature at which molecular mobility begins to take place and below which molecular mobility is frozen and the elastomer becomes rigid and glassy. Density data from dynamic equilibrium simulations at 21 specified temperature points for the extracted asphalt and the MDI-modified asphalt were used to plot the temperature–density variation curves. The curves on both sides of the inflection point were fitted separately, and the temperature corresponding to the intersection of the curves represents the glass transition temperature of the two types of asphalt, as shown in Figure 11. From the fitted curve changes in the graph, it was determined that at a crosslinking degree of 90% the glass transition temperature of the base asphalt is approximately 390 K, while that of the MDI-modified asphalt is around 425 K.

Figure 11 shows that the glass transition temperature of MDI is higher than that of regular asphalt. The introduction of MDI as a modifier in asphalt results in changes in the molecular structure, enhancing the mutual attraction between molecules or altering the arrangement of molecules. It improves the stability of the material, maintaining a higher glass transition temperature at elevated temperatures. A higher glass transition temperature signifies that the MDI-modified asphalt is more stable at high temperatures and less prone to softening or losing strength. Therefore, MDI-modified asphalt may retain a higher elasticity at lower temperatures than regular asphalt, while maintaining high-temperature stability in hot climates.

## 4. Asphalt Mixture Performance

**Figure 12:**
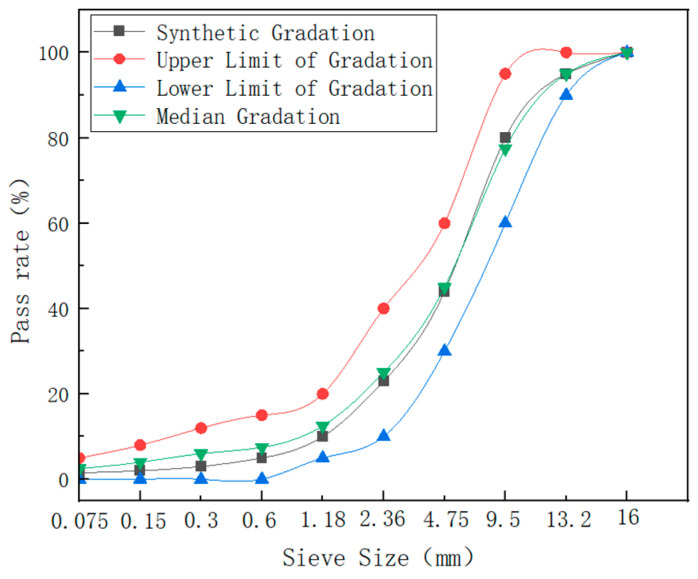
Gradation curve of the LB-13 CMA mixture.

According to the Technical Specifications for Construction of Highway Asphalt Pavements (JTG F40-2004) [35], the gradation LB-13 was selected to design the cold-mixed asphalt (CMA) mixture shown in Table 5. The synthetic gradation used in this study falls within the upper and lower limits specified in the technical specification and closely approximates the median region. The designed gradation curve is shown in Figure 12.

### 4.1. Marshall Stability

In order to assess the performance of the new modified asphalt mix (CMA), Marshall stability and flow tests were conducted. The stability and flow properties were tested for the MDI-modified asphalt mixture with a 15% content, as well as for the MDI-modified asphalt mixtures (15% MDI, 15% rock asphalt) with diluent contents of 10% and 15%. The results are presented in Table 6.

**Figure 13:**
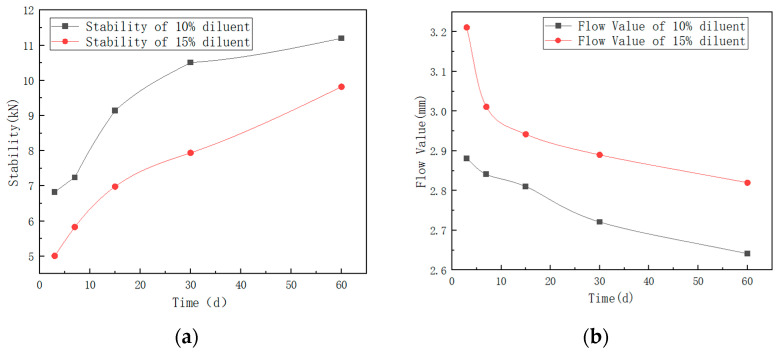
Stability and flow value variations over time for two different dilution levels. (**a**) Stability and (**b**) flow values.

Due to the volatilization of the diluent requiring some time, the strength of the asphalt mix gradually increased over time. Long-term curing tests were then conducted on the MDI-modified asphalt mixes with two different diluent content levels. The stability and flow value test results at different curing times are listed in Table 7 and the curves are also plotted in Figure 13a,b.

From Figure 13, it is evident that the stability of the 10% diluent is notably higher over time compared to that of the 15% diluent. Additionally, the flow value of the 10% diluent is significantly lower than that of the 15% diluent. According to the specifications in the Technical Specifications for Construction of Highway Asphalt Pavements (JTG F40-2004) [35], the Marshall stability should not be less than 3 kN, and the flow value should be within the range of 2–4 mm. These test results comply with the specified requirements. This study primarily focuses on the use of asphalt in the construction and maintenance of high-grade roads. However, there are no specific regulations in the standards regarding this matter. Therefore, referring to the specifications for hot-mixed asphalt (AC), it is stipulated that the Marshall stability for expressways should be ≥8, while for other road surfaces, it should be ≥5. The results indicate that after 10 days of molding, the Marshall stability of the asphalt mixture with 10% MDI modification and diluent meet the specifications for expressways. With time, the asphalt mixture with 15% MDI modification and diluent also meet the requirements. In general, the Marshall stability values gradually increased over time, while the flow values generally decreased.

### 4.2. High-Temperature Performance

Rutting is a common form of pavement defect mainly caused by softening of the asphalt pavement and subsequent plastic deformation under high temperatures and a high density of traffic. The formation of rutting causes serious harm to road driving; thus, it is crucial to ensure asphalt pavements possess high temperature susceptibility. This study uses dynamic stability tests (also known as rutting tests) to evaluate the high-temperature performance of the prepared CMA asphalt mixture [36].

Using the CMA asphalt mix LB-13, rutting test specimens were prepared. Considering the significant impact of curing time on the strength development of CMA, the specimens were tested after 7 days of curing. The results for dynamic stability (DS) obtained in this study are presented in Table 8. It is observed that the dynamic stability value of the cold-mixed MDI-modified asphalt is 1568 cycles/mm, far exceeding the 800 cycles/mm specified for hot-mixed asphalt AC in the Technical Specifications for Construction of Highway Asphalt Pavements (JTG F40-2004) [35]. This indicates that the performance of the newly developed cold-mixed asphalt mixture meets the performance requirements for high-grade pavements.

### 4.3. Water Damage Resistance

In China, a significant portion of asphalt pavement defects are caused by water infiltrating into the structural layers of the pavement. Moisture diminishes the adhesion and strength of the asphalt mix, leading to the gradual emergence of various pavement issues. This study conducted immersion Marshall tests to evaluate the water damage resistance of the prepared CMA mixture, and the results are listed in Table 9. From these results, it can be observed that the residual stability of the MDI-modified cold-mixed asphalt is 87, surpassing the standard value for HMA (AC) outlined in the Technical Specifications for Construction of Highway Asphalt Pavements (JTG F40-2004) [35]. This indicates that the newly developed cold-mixed asphalt mixture exhibits enough water stability performance for asphalt pavement, and is not less than the hot-mixed asphalt AC.

## 5. Conclusions

This study addresses the performance issues of cold-mixed asphalt mixtures by preparing MDI-modified asphalt based on molecular dynamics simulations and road performance analyses of asphalt mixtures. The following conclusions can be drawn:

(1) Due to the potential decrease in the softening point and compromised high-temperature performance caused by the addition of MDI, rock asphalt was employed for further improvement. Following tests and analyses of various blends of MDI-modified asphalt with different incorporation levels of rock asphalt, a mixture of 15% rock asphalt and 15% MDI-modified asphalt demonstrated a more balanced overall performance.
(2) Utilizing Materials Studio software, an MDI-modified asphalt model was simulated, and its fundamental properties, such as density, free volume, cohesive energy density, and glass transition temperature, were evaluated. The results indicate that the MDI-modified asphalt exhibits enhanced resistance to compression, durability, and cracking, and stability. These improvements contribute to enhanced curing performance, ultimately improving pavement stability and longevity, thereby reducing pavement damage.
(3) Taking LB-13 gradation as an example, MDI-modified cold-mixed asphalt was prepared. The mechanical properties, high-temperature performance, and resistance to water damage were tested according to China’s specifications. The results indicate that the stability of the MDI-modified cold-mixed asphalt prepared here exceeds the requirements for hot-mixed asphalt (AC) specified in the Chinese standards. High-temperature rutting tests and resistance to water damage demonstrated that the prepared MDI-modified cold-mixed asphalt performs notably better in resisting rutting at high temperatures and water damage, consistent with the hot-mixed asphalt mixture.

## Figures and Tables

**Table 1:** Technical properties of petroleum asphalt.

Technical Properties	Experimental Value	Test Method (JTG E20-2011)
Penetration value, 1/10 mm (25 °C, 100 g, 5 s)	80.7	T0604
Softening point, °C (R&B)	47	T0606
135 °C Kinematic viscosity, mPa·s	346.3	T0619
5 °C Ductility, mm	89	T0605

**Table 2:** The technical properties of rock asphalt.

Testing Items	Indicators
Natural asphalt content, %	28 ± 3
Trichloroethylene solubility, %	25~31
Density, g/cm^3^	1.42
Flash point, °C	≥230
Heating loss, %	<1.2
Moisture content,%	<2
Maximum particle size, mm	<0.3

**Table 3:** Modified asphalt component mass fractions and functions.

Raw Material	Mass Fraction	Function
Asphalt	282 g	-
MDI	24 g	Modification plasticizer
Petroleum Resin	20 g	Enhances the flexibility of asphalt
Setting Accelerator	4 g	Increases bonding strength
Ethylene Glycol	40 mL	Improves compatibility with other components
Kerosene	40 mL	Dilutes asphalt
Cellulose	6 g	Enhances stability of the mixture

**Table 4:** Technical properties of MDI-modified asphalt.

Item	Penetration Value (mm) (25 °C, 100 g, 5 s)	Softening Point (°C)	5 °C Ductility (mm)
10% MDI + petroleum asphalt	-	43.9	140
15% MDI + petroleum asphalt	-	36.8	180
20% MDI + petroleum asphalt	-	14.8	210
10% rock asphalt + petroleum asphalt	65	49.7	47
15% rock asphalt + petroleum asphalt	55	54.7	38
20% rock asphalt + petroleum asphalt	52	57.4	25
10% rock asphalt + 15% MDI-modified asphalt	137	34	219
15% rock asphalt + 15% MDI-modified asphalt	83	48.4	67.5
20% rock asphalt + 15% MDI-modified asphalt	76	49.9	44
Test method (JTG E20-2011)	T0604	T0606	T0605

**Table 5:** Synthetic gradation of LB-13.

Item	Cumulative Passing Rates at Different Sieve Sizes (mm)									
	16	13.2	9.5	4.75	2.36	1.18	0.6	0.3	0.15	0.075
Synthetic Gradation (%)	100	95	80	44	23	10	5	3	2	1.5
Upper Limit of Gradation (%)	100	100	95	60	40	20	15	12	8	5
Lower Limit of Gradation (%)	100	90	60	30	10	5	0	0	0	0
Median Gradation (%)	100	95	77.5	45	25	12.5	7.5	6	4	2.5

**Table 6:** Test results of MDI-modified CMA mixtures.

Item	Stability (kN)	Flow Value (mm)
MDI-Modified Asphalt Mixture with 15% MDI + 0% Rock Asphalt + 0% Diluent	4.23	3.670
MDI-Modified Asphalt Mixture with 15% MDI + 15% Rock Asphalt + 10% Diluent	8.46	2.251
MDI-Modified Asphalt Mixture with 15% MDI + 15% Rock Asphalt + 15% Diluent	6.22	2.791
Required Value for CMA (Low-Grade Highway)	>3	2–4
Required Value for HMA (AC)	≥8 (Expressway); ≥5 (Others)	2–4.5
Test method (JTG E20-2011)	T0709	

**Table 7:** Marshall test results of diluent mixtures.

Item		Time				
		3 Days	7 Days	15 Days	30 Days	60 Days
MDI-Modified Asphalt Mixture with 15% MDI + 15% Rock Asphalt + 10% Diluent	Stability (kN)	6.82	7.24	9.14	10.51	11.20
	Flow Value (mm)	2.881	2.841	2.810	2.721	2.641
MDI-Modified Asphalt Mixture with 15% MDI + 15% Rock Asphalt + 15% Diluent	Stability (kN)	5.01	5.83	6.98	7.94	9.82
	Flow Value (mm)	3.211	3.011	2.942	2.890	2.820

**Table 8:** Dynamic stability of the CMA mixture.

Item	Dynamic Stability (60 °C, 0.7 MPa) (Cycles/mm)
MDI-Modified Asphalt Mixture with 15% MDI + 15% Rock Asphalt + 10% Diluent	1568
Required value for HMA (AC)	≥800
Test method (JTG E20-2011)	T0719

**Table 9:** Residual stability results of immersion Marshall tests.

Item	Residual Stability
MDI-Modified Asphalt Mixture with 15% MDI + 15% Rock Asphalt + 10% Diluent	87
Standard value for HMA (AC)	≥80
Test method (JTG E20-2011)	T0709

## Data Availability

Data are contained within the article.
